# Bayesian prediction of placebo analgesia in an instrumental learning model

**DOI:** 10.1371/journal.pone.0172609

**Published:** 2017-02-22

**Authors:** Won-Mo Jung, Ye-Seul Lee, Christian Wallraven, Younbyoung Chae

**Affiliations:** 1 Acupuncture & Meridian Science Research Center, College of Korean Medicine, Kyung Hee University, Seoul, Republic of Korea; 2 Department of Brain Cognitive Engineering, Korea University, Seoul, Republic of Korea; Radboud University Medical Centre, NETHERLANDS

## Abstract

Placebo analgesia can be primarily explained by the Pavlovian conditioning paradigm in which a passively applied cue becomes associated with less pain. In contrast, instrumental conditioning employs an active paradigm that might be more similar to clinical settings. In the present study, an instrumental conditioning paradigm involving a modified trust game in a simulated clinical situation was used to induce placebo analgesia. Additionally, Bayesian modeling was applied to predict the placebo responses of individuals based on their choices. Twenty-four participants engaged in a medical trust game in which decisions to receive treatment from either a doctor (more effective with high cost) or a pharmacy (less effective with low cost) were made after receiving a reference pain stimulus. In the conditioning session, the participants received lower levels of pain following both choices, while high pain stimuli were administered in the test session even after making the decision. The choice-dependent pain in the conditioning session was modulated in terms of both intensity and uncertainty. Participants reported significantly less pain when they chose the doctor or the pharmacy for treatment compared to the control trials. The predicted pain ratings based on Bayesian modeling showed significant correlations with the actual reports from participants for both of the choice categories. The instrumental conditioning paradigm allowed for the active choice of optional cues and was able to induce the placebo analgesia effect. Additionally, Bayesian modeling successfully predicted pain ratings in a simulated clinical situation that fits well with placebo analgesia induced by instrumental conditioning.

## Introduction

The placebo effect has been regarded as a conjunction of automatic conditioning effects and cognitive expectancy effects based on conscious contextual information [1]. People can learn to obtain benefits through verbally induced expectations, cued and contextual conditioning, and/or observational and social learning [2]. Accordingly, these types of learning processes guide changes in behavior and expectations that could lead to the formation of placebo analgesia [3,4]. Many studies have found that experimental conditioning induces placebo analgesia [5–9]; most of these studies implemented conditioning using Pavlovian conditioning paradigms that involved hard-wired passive learning of relations among events (i.e. the learning of conditioned stimulus to unconditioned stimulus associations), making it possible to predict and avoid the occurrence of potentially harmful stimuli. These paradigms, however, do not consider the learners’ actions, that is, whether intentional exploration or behavior changes will influence the learning outcome [10].

On the other hand, instrumental conditioning involves action that is taken according to certain cues, and allows for the possibility of choices. This enables learning of the actions and their consequences, which may either be rewards or punishments [11]. During instrumental conditioning procedures, the brain provides a common currency for decision-making that incorporates reward acquisition and punishment avoidance [12]. While there is ample evidence that the association of the events in Pavlovian conditioning is valid in the real world [13], the action and behavior changes that are involved in instrumental learning may be more similar to an actual clinical setting. Thus, it is important to consider the capacity of an individual to assess potential rewards based on the outcome of pain relief in real world settings [14]. In instrumental learning (operant learning) paradigms, the brain computes the error between predicted and actual outcomes and uses that error value to improve future predictions and actions [15]. In the context of placebo analgesia, previous studies have utilized animal models together with instrumental conditioning for understanding the development of placebo analgesia [16]. However, to the best of our knowledge, no human studies have so far investigated placebo analgesia using an instrumental learning model.

According to the framework of predictive coding, the brain actively makes inferences based on prior experiences and expectations [17]. An inferential process in the brain is conceptualized as perception, in which information from prior experiences is used to generate expectations about future perception and to interpret sensory inputs [18]. Within the Bayesian theoretical and mathematical framework, the brain constantly interprets sensory inputs through the method of minimizing the average of prediction errors across the whole sensory system [19]. Thus, employing a Bayesian framework of brain function would benefit the current understanding of placebo analgesia in instrumental conditioning paradigms. Placebo analgesia is then regarded as a probabilistic integration between top-down expectations of prior pain and bottom-up sensory signals [17]; from this perspective, it is important to consider not only the averaged magnitude of previous pain experiences but also the precision of the constructed expectation. Recently, a computational investigation of pain supported the idea that the Bayesian model reflects the strategies used during pain perception by showing that modulation due to disparate factors is intrinsic to the pain process [20]. However, no studies have attempted to predict the placebo response during instrumental conditioning using such a predictive coding framework.

Thus, in the present study, an instrumental learning model was implemented by adapting a trust game to a clinical situation, in which two available options, labeled “doctor” and “pharmacy”, were associated with different magnitudes (intensity) and degrees of precision (variation) of pain. This simulation resembles a real-world situation and the participants were allowed to explore, experience, and evaluate the two options actively. This experimental paradigm was used to investigate whether the placebo analgesic responses to behavioral actions were associated with less pain. Furthermore, Bayesian modeling was applied to predict the placebo responses of the participants within the instrumental conditioning model.

## Methods

### Participants

This study included 24 healthy human volunteers (14 males, 10 females) who were recruited through an advertisement. None of the participants had any history of neurological, psychiatric, or other major medical problems and none were using medications at the time of the study. There were no drop-outs after the initial inclusion in the experiment. Each participant received a detailed explanation of the study, and written informed consent was obtained prior to participation. All procedures were performed with the approval of the institutional review board of Korea University, Seoul, Republic of Korea.

### Experimental design and procedure

To assess placebo analgesia, a computerized task mimicking the medical decision-making process was implemented; this task was called a “medical trust game” inspired and modified from a previously-published economic trust game [21,22]. Prior to the experiment, participants were informed that this experiment was designed to measure the treatment utilization tendencies of each participant through computerized experiments. Thus, participants played a medical trust game in which a hypothetical situation of visiting a doctor to relieve pain was instituted.

In each trial, participants made a series of 40 trust-related decisions that each involved the presentation of a different face on the screen; they were given 2,000 Korean won (approximately $2 USD) for each trial. All of the faces were Asian, between 25 and 50 years of age, photographed directly from the front with the subject wearing a white coat, and presented in greyscale. Upon seeing the doctor, each participant decided whether they would receive care from this doctor (with payment of $2) or take a pill from a pharmacy (with payment of $1). The participants were informed that they would receive a small percentage of their overall game earnings in addition to a fixed compensation amount to be paid after the experimental task. The trials were programmed using the Psychtoolbox program in Matlab (MathWorks, Natick, MA, USA).

Each participant was informed that the face of a doctor would be shown on the screen after exposure to the first painful stimuli (the reference pain: 512 mN [high intensity pain]) inflicted with a weighted needle (PINPRICK stimulators; MRC Systems, Heidelberg, Germany) [23,24]. The administration of the pain stimulus was hidden by a panel on the left-hand side of the participant; the stimulus was applied to the back of the left hand between the index finger and thumb while the participant’s hand formed a loose fist. A perceived pain rating was obtained using the “magnitude estimation” method [25], in which participants rate the pain intensity of the second stimulus relative to that of the first fixed stimulus (i.e., the reference pain).

**Fig 1:**
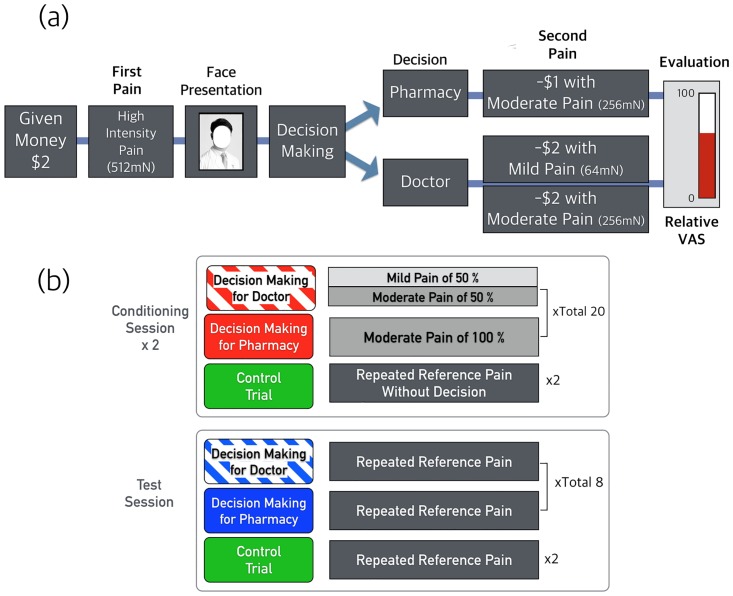
(a) Experimental procedures: A computerized medical trust game. (b) During the conditioning session, choosing treatment from a doctor (with 2 $ cost) resulted in reduced secondary pain, just as in the conventional trust game; the reduction was either large (from 512 mN to 64 mN) or small (512 mN to 256 mN) based on a 50% probability (low precision). Choosing pills from a pharmacy (with 1 $ cost) resulted in a small pain reduction (512 mN to 256 mN) with a 100% probability (high precision). The task was repeated 40 times during the conditioning procedure. During the test session, a high degree of pain (the intensity of reference pain, 512 mN) was always delivered as the secondary pain.

The instructions covered two options: 1) choose to receive a pill from a pharmacy, which would reduce their pain to a fixed moderate level (256 mN), or 2) choose to be treated by the doctor, which would reduce their pain to either a mild level (64 mN), or to the same moderate level (256 mN) as the medicine. Following the decision, a decreased amount of pain was delivered during the conditioning session. Participants were asked to evaluate the effectiveness of the treatment from either a doctor or the pharmacy compared to the reference pain [24]. Thus, in the medical trust game, the participants had to decide between choosing a doctor or choosing the pharmacy, based on the tradeoff between pain relief from a doctor and cost of payment. This tradeoff allowed for distribution of both choices in decisions. During the conditioning session, choosing treatment from a doctor resulted in a reduction in the degree of the secondary pain, just as in the conventional trust game; the decrease of pain was either large (from 512 mN to 64 mN) or small (512 mN to 256 mN) based on a 50% probability (low precision). Choosing pills from a pharmacy resulted in a small degree of pain reduction (512 mN to 256 mN) with a 100% probability (high precision). The task was repeated 40 times during the conditioning procedure. In contrast, during the test session, a high degree of pain (intensity of reference pain, 512 mN) was always delivered as the secondary pain (Fig 1).

### Prediction of placebo response using Bayesian modeling

Placebo analgesia was modeled by constructing a Bayesian framework (Eq 1), in which the pain ratings corresponded to a posterior distribution, and by fitting a logarithmic relationship between pain intensity and pain rating as the likelihood. Using this Bayesian framework, the placebo response can be modeled as an inferential response according to the discrepancy between the ascending sensory signal and the descending prediction of pain. It is assumed that the subjective pain rating can be predicted based on the posterior probabilistic distribution of the Bayesian inference.

For the model predictions, free variables from the Bayesian model were trained based on data from the conditioning session and used to predict the posterior probability of pain ratings being at the same intensity as the reference pain using the fitted model. Thus, test session data were not used in the training to predict the pain ratings of the test session. The intensity of sensory input, or the pain stimulation given through PINPRICK device, was parameterized in the perceptual dimension. In this process, the logarithmic relationship between pain intensity and the weight of the PINPRICK device was calculated based on a previous study (Eq 3) [24].

The formulated Bayesian model used in the present study is described below:

Pr(P|C,S)∝Pr(P|C)×Pr(S|P) (1)

$$\text{Pr}\left(P|C\right)\sim Normal\left(\mu_{prior},\sigma_{prior}\right)$$ (2)

$$\text{Pr}\left(S|P\right)\sim Normal\left(\mu_{s}\propto \left(a_{i}\times \text{log}\left(weight\right)+b_{i}\right),\sigma_{s}\right)$$ (3)

$$\text{Pr}\left(P|C,S\right)\sim Normal\left(\mu_{post},\sigma_{post}\right),$$ (4)

*where*

$$\;\mu_{post}=\left(\frac{\mu_{s}}{\sigma_{s}{}^{2}}+\frac{\mu_{prior}}{\sigma_{prior}{}^{2}}\right)\times \left(\frac{\sigma_{s}{}^{2}+\sigma_{prior}{}^{2}}{\sigma_{s}{}^{2}\times \sigma_{prior}{}^{2}}\right)$$

$$\frac{1}{\sigma_{post}{}^{2}}=\frac{1}{\sigma_{prior}{}^{2}}+\frac{1}{\sigma_{s}{}^{2}}$$

P: pain perception, C: condition (decision for doctor or pharmacy), S: pain stimulation

The prior distribution in each decision-making situation was estimated from the fitted Bayesian model using the Markov chain Monte Carlo (MCMC) algorithm. The PyMC Python library was used for the model fitting and the estimation (https://pymcmc.readthedocs.org/en/latest). Based on the fitted prior probabilistic distribution, the pain rating in the test procedure was predicted and compared to the actual reported pain in the test session [26].

We compared our model with linear regression model, a possible alternative for analysis in this study. In the linear regression model, we used the same logarithmic relationship between the pain intensity and the weight of the PINPRICK as used in the Bayesian model. We studied different linear regression models based on the conditioning session data, depending on whether the choice was doctor or pharmacy in individual participants. The linear regression has one explanatory variable, the estimated intensity of sensory input from given PINPRICK weight, and one response variable, the pain rating from the participant. For model comparison, the trained models with conditioning session data were applied to predict pain ratings in the testing session. The Bayesian information criterion (BIC) was calculated based on a models’ prediction error on the testing session data—a lower BIC value indicates a better explanation.

## Results

### Choice probabilities and pain experience during the medical trust game

**Fig 2:**
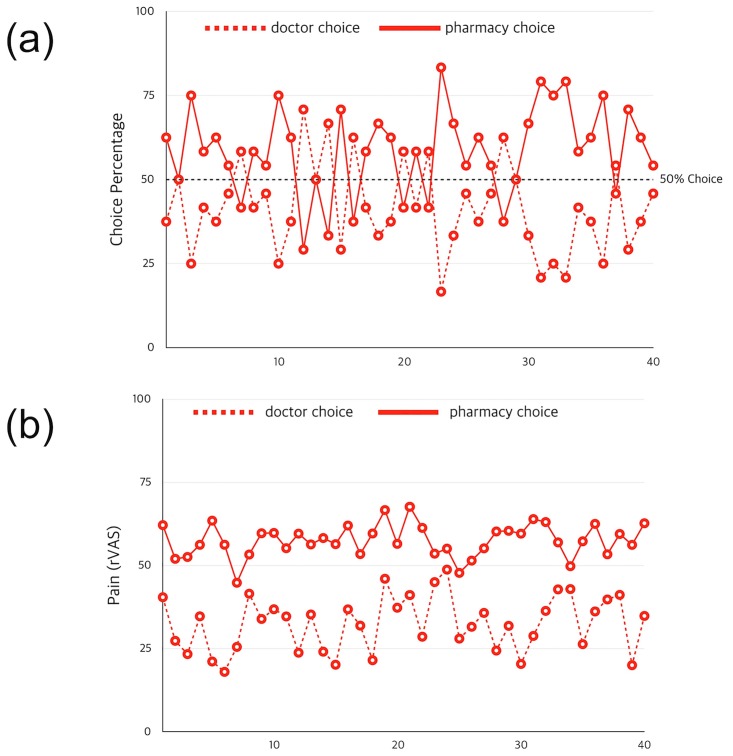
(a) Changes in choice probabilities during the conditioning period of the medical trust game. The choice probabilities between the doctor and the pharmacy ranged from 16.7–83.3% over 40 trials and, on average, participants selected the doctor option 40.3% of the time and the pharmacy option 59.7% of the time. (b) Changes in pain experience during the conditioning period of the medical trust game; on average, the participants experienced pain after both the doctor (32.5 ± 1.3) and pharmacy (57.5 ± 0.8) choices. The red solid line represents the decision for pharmacy treatment during the conditioning period and the red dotted line represents the decision for doctor treatment during the conditioning period.

During the conditioning period of the medical trust game, participants made decisions regarding whether to receive pain treatment from a doctor (40.3%) or from the pharmacy (59.7%). The choice probabilities between the doctor and pharmacy ranged from 16.7–83.3% over the 40 trials; there were no significant changes in the choice probabilities over time. During the conditioning period of the medical trust game, participants experienced pain after both the doctor choice (32.5 ± 1.3%) and the pharmacy choice (57.5 ± 0.8%). The pain rating after the doctor choice ranged from 18.0–48.8, and the pain rating after the pharmacy choice ranged from 44.8–67.6 over the 40 trials (Fig 2).

### Placebo response during instrumental conditioning

The magnitude, or intensity, of pain—according to the ratings of the participants—were compared with the test session. Compared to the control trials, significantly lower pain ratings were reported when the doctor was chosen for treatment (84.6 ± 2.2 vs. 92.8 ± 2.2, degrees of freedom [df] = 23, *t* = 3.308, *p* < 0.01) and when the choice to receive pills was made (87.6 ± 1.9 vs. 92.8 ± 2.2, df = 23, *t* = 2.209, *p* < 0.05). However, the pain ratings between the doctor choice and the pharmacy choice did not differ significantly (84.6 ± 2.2 vs. 87.6 ± 1.9, df = 23, *t* = 1.340, *p* = 0.193).

**Fig 3:**
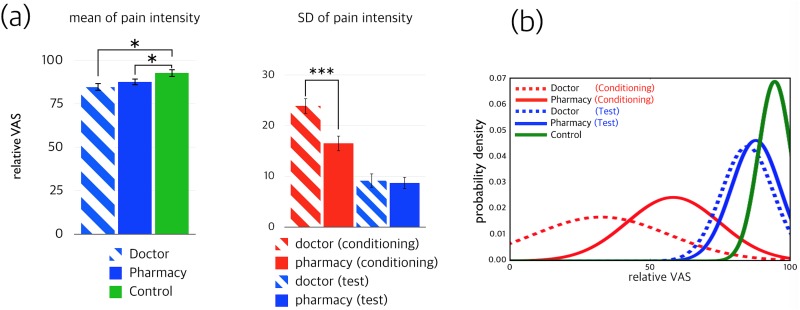
(a) Means and standard deviations (SD) of the pain intensities in the two different placebo analgesia groups. Significantly less pain was experienced following the decision for doctor treatment than during the control trial in the test session. Likewise, significantly less pain was experienced following the decision for pharmacy treatment than during the control trial in the test session. However, there were no significant differences between the decision for doctor treatment and the decision for pharmacy treatment. In terms of the SD, the choice for doctor treatment had significantly larger SDs compared to the choice for pharmacy treatment during the conditioning session, while no significant difference in variance was found between the doctor and pharmacy choices during the test session. (b) Reconstructed normal distributions of pain ratings in the conditioning and test sessions using the average of individual mean and SD values. The red dotted line represents the decision for doctor treatment during conditioning and the red solid line represents the decision for pharmacy treatment during conditioning. The green solid line represents control trials. The blue dotted line represents the decision for doctor treatment during testing and the blue solid line represents the decision for pharmacy treatment during testing.

To determine precision, or certainty, the individual standard deviation (SD) values between conditions for each participant were compared using paired *t*-tests for both the conditioning and test sessions. The SD values of the pain ratings following the doctor choice and the pharmacy choice during the conditioning session differed significantly (23.9 ± 1.5 vs. 16.5 ± 1.5, df = 23, *t* = 6.479, *p* < 0.001). Regardless of the large variance in pain ratings between the two choices during the conditioning session, there were no significant differences in variance between the doctor and pharmacy choices during the test session (Fig 3).

### Predictions of placebo response using Bayesian modeling

**Fig 4:**
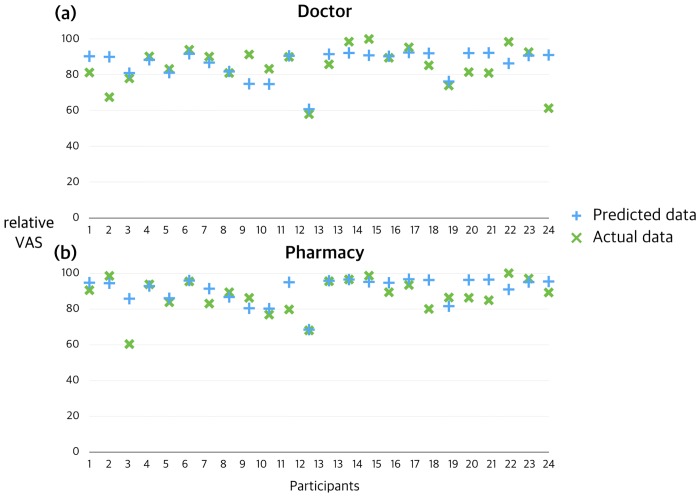
Predictions of placebo response using Bayesian modeling. Model-predicted pain ratings and actual pain ratings of the individual participants during the test session. Significant correlations exist for both choices. (a) Decision for doctor; (b) Decision for pharmacy. Blue +: predicted data, Green ×: actual data.

The predicted pain ratings and actual reports of pain from participants during the test session were significantly correlated for both the doctor and pharmacy choices (Fig 4). For the prediction using the Bayesian model, the decision to be treated at a pharmacy conditioned with high precision showed a more reliable prediction (BIC = 126.85, root-mean-square error [RMSE] = 8.40) than the decision to be treated by a doctor conditioned with low precision (BIC = 131.34, RMSE = 10.12). In the model comparison, the predictions of the Bayesian model were superior to the predictions of the linear regression model (Pharmacy: BIC = 156.21, RMSE = 22.46; Doctor: BIC = 165.77, RMSE = 28.45).

## Discussion

The present study utilized a medical trust game in which the choice of treatment for pain from either a doctor or a pharmacy was offered; the decision between the two choices was associated with pain relief after a monetary payment. In the test session, participants reported a lower degree of pain intensity when they chose either of the two treatment choices compared to the control condition. This result indicates that placebo analgesia was successfully induced through an instrumental learning paradigm, and suggests that prior experience and/or expectations successfully influenced the perception of pain when active exploration and evaluation were allowed.

The efficacy of medical treatment is critically determined by treatment history [27]. For example, secondary treatment produces a significantly greater degree of pain reduction in patients with a positive treatment history [28]. Likewise, prior experience modulates the efficacy of a subsequently applied active treatment by altering treatment-specific expectations, either through conditioning or a combination of both expectation and conditioning mechanisms [29]. In the present study, the choice probabilities between the doctor and the pharmacy ranged from 16.7–83.3% over 40 trials; overall, participants chose the doctor 40.3% of the time (more pain relief but with high cost) and the pharmacy 59.7% of the time (less pain relief but with low cost). Because there were two different levels of pain reduction that the participants experienced upon choosing the doctor, they exhibited greater variations in their pain ratings over the conditioning period of the medical trust game. During the game, the participants had to decide between a doctor or a pharmacy based on the tradeoff between pain relief and the monetary cost of payment. Based on this decision-making process, the participants were able to learn the associations between their choices and their experience of pain relief. Under these conditions, it can be expected that people will call upon their treatment histories, with respect to doctors and pharmacies, to establish expectations for treatment in an experimental setting.

Instrumental learning optimizes future decisions based on the consequences of those decisions. Thus, instrumental conditioning can efficiently shape reward/punishment values in humans, even when the information related to consequences is delivered in a subliminal manner [30]. The concept of “making a decision” critically distinguishes instrumental learning from Pavlovian learning because, compared to the latter, an organism not only decides its behavior or action, but is also sensitive to the consequences of that behavior or action, in the instrumental learning paradigm. Furthermore, there is evidence that information from Pavlovian conditioning is transferred to instrumental conditioning, strengthening the learning from instrumental conditioning, while behavioral responses provide properties that can act as conditioned stimuli in Pavlovian conditioning, suggesting for an interaction between the two forms of conditioning [11,31,32]. Thus, instrumental conditioning allows for exploration of possible choices, as well as control of future consequences after learning is accomplished. This study illustrates the exploration of possible treatments between the doctor and pharmacy according to the evaluation of the two choices under different conditions. Substantial evidence indicates that the controllability of upcoming pain diminishes pain intensity [33–35] and it has been shown that pain and economic value can be integrated in a cost-benefit equation that informs the decision-making process [36]. Despite the importance of instrumental conditioning in pain research, to the best of our knowledge, no studies have so far investigated placebo analgesia induced by instrumental conditioning. Thus, the present results may be important for the implementation of instrumental conditioning paradigms in the field of placebo analgesia research.

In the present study, there were significant differences in pain ratings following the choice of either a doctor or a pharmacy in the conditioning session (32.6 ± 2.5 for doctors vs. 58.2 ± 2.6 for pharmacy, *t* = 9.926, *p* < 0.0001). Similar pain reductions were observed following the choice of either a doctor or a pharmacy in the test session. The Bayesian formulation within the predictive coding framework can directly account for differences in the magnitude and precision of expectations, which are highly associated with the strength of the placebo response [17]. Predictive coding suggests that probabilistic representations act as top-down influence on expectations explaining away bottom-up prediction errors between expected and actual sensory events [37]. From the perspective of predictive coding, an active inference is carried out by fulfilling predictions through actions based on perceived inference or expectation [38]. Recently, it was reported that the socially-acquired uncertainty of upcoming pain alleviates the subjective rating of painful stimulation [39]. In the present study, in terms of precision, choosing a doctor was associated with pain relief that had more uncertain expectations (SD = 23.9 ± 1.5) than choosing a pharmacy (SD = 16.5 ± 1.5). Thus, in agreement with Bayesian integration, pain relief with greater uncertainty was perceived as being of a lower degree when choosing a doctor, even with the expectation of greater pain relief.

Models based on Bayesian theory have been successfully applied to explain the perceptions arising from the integration of top-down and bottom-up information in the fields of vision and touch perception [40–42]. The concept of placebo analgesia, in which prior information about the context biases pain perception, also ties in well with the Bayesian framework [17]. For example, it was demonstrated that hierarchical Bayesian modeling fits well with experimental pain data [20]. In the present study, it was possible to successfully predict pain ratings in the test session by fitting a model using data from the conditioning session, which implies that the Bayesian framework fits well with placebo analgesia induced by instrumental conditioning. Because actions and/or behaviors are essential constituents of the Bayesian brain, the Bayesian model used in the present study, of placebo analgesia induced by instrumental conditioning, may provide an important link between placebo analgesia and the Bayesian brain.

The present study had several limitations. First, the prior experiences of a participant with a doctor or pharmacy could have influenced their placebo responses while they were deciding between these two options. However, even though these possible confounding factors were not fully excluded from the present experiment, a more realistic situation in which a medical decision was needed from each participant was utilized. Second, a placebo response following Pavlovian conditioning was not assessed in this study. It would be interesting to compare the placebo responses between instrumental conditioning and Pavlovian conditioning in future studies. Third, the sample size of twenty-four is relative small to be interpreted in the general population. Therefore, a replication of this study within a larger study population is warranted. Finally, the sequential order of learning was not considered as a variable in the present model and, because optimizing the decision model by updating information associated with action on a trial by trial basis likely occurred, the sequential timing might have been a practical element to model instrumental learning.

In sum, the instrumental learning paradigm used in the present study allowed for the active choice of optional cues that were able to induce the placebo analgesia effect. Additionally, placebo responses were predicted based on decision-making across individuals using a Bayesian model. Thus, the present findings allow for a more comprehensive view of placebo analgesia that embraces active decision-making, which almost certainly occurs in real world settings.
